# Quality by design optimization of microemulsions for topical delivery of *Passiflora setacea* seed oil

**DOI:** 10.3762/bjnano.16.146

**Published:** 2025-11-20

**Authors:** Daniel T Pereira, Douglas Dourado, Danielle T Freire, Dayanne L Porto, Cícero F S Aragão, Myla L de Souza, Guilherme R S de Araujo, Ana Maria Costa, Wógenes N Oliveira, Anne Sapin-Minet, Éverton N Alencar, Eryvaldo Sócrates T Egito

**Affiliations:** 1 Graduate Program in Health Sciences, Federal University of Rio Grande do Norte (UFRN), R. Gen. Gustavo Cordeiro de Farias, s/n, Natal-RN, 59012‑520, Brazil; 2 CITHEFOR, Université de Lorraine, Nancy, F-54000, France; 3 Laboratory of Micro and Nanostructured Systems, College of Pharmaceutical Sciences, Food and Nutrition, Federal University of Mato Grosso do Sul (UFMS), Cidade Universitária, s/n, Campo Grande-MS, 79070-900, Brazil; 4 Department of Pharmacy, Federal University of Rio Grande do Norte, R. Gen. Gustavo Cordeiro de Farias, s/n, Natal-RN, 59012-570, Brazil; 5 Laboratório de Tecnologia dos Medicamentos, Department of Pharmaceutical Sciences, Federal University of Pernambuco (UFPE), Av. Prof. Moraes Rego, 1235, Recife-PE, 50670-901, Brazil; 6 Department of Pharmaceutical Sciences, Federal University of Sergipe (UFS), Av. Marcelo Deda Chagas, s/n, São Cristóvão-SE, 49100-000, Brazil; 7 Embrapa Cerrados, Laboratory of Food Science, BR-020, km 18, s/n, Planaltina-DF, 73310-970, Brazil

**Keywords:** design of experiments (DoE), microemulsions, nanotechnology, natural products, passion fruit, quality by design, skin delivery

## Abstract

*Passiflora setacea* seed oil is a natural source of bioactive unsaturated fatty acids, notably linoleic acid (ω-6) and oleic acid (ω-9), with promising antioxidant and anti-inflammatory potential for dermatological applications. However, its direct use is limited by poor physicochemical and organoleptic properties. This study aimed to develop and optimize a topical microemulsion (ME) system incorporating *P. setacea* seed oil using quality by design principles to address formulation challenges. The oil was extracted via Soxhlet and characterized by gas chromatography–mass spectrometry and thermal analysis. A full factorial design, followed by a Box–Behnken design, was employed to optimize the formulation based on critical quality attributes and the defined quality target product profile. The optimized ME presented a hydrodynamic diameter of approximately 22 nm and polydispersity index below 0.2 and remained stable for 60 days. The ME was gelled with sodium carboxymethyl cellulose, while vitamin E and Liquid Germall^®^ Plus were incorporated as antioxidant and preservative agents, respectively, yielding the final topical gel formulation. Cytocompatibility assays demonstrated high cell viability for ME at concentrations below 2 mg/mL in RAW 264.7 macrophages and 0.5 mg/mL in human umbilical vein endothelial cells. Overall, this work presents a promising nanotechnology-based topical delivery platform for *P. setacea* seed oil, employing quality by design principles to ensure formulation performance, stability, and skin cell compatibility.

## Introduction

Species of the *Passiflora* genus are known for their rich composition of fixed oils and bioactive compounds, including flavonoids and alkaloids, which exhibit significant therapeutic potential [1]. While *Passiflora edulis*, *P. alata*, and *P. incarnata* have been extensively studied in phytopharmaceutical research, recent investigations have turned attention toward underexplored wild species, such as *Passiflora setacea* [2–3].

Notably, seed oil extracted from *P. setacea* (OPS) is particularly rich in unsaturated fatty acids, predominantly linoleic acid (ω-6) and oleic acid (ω-9). Compared to other wild *Passiflora* species, OPS exhibits a markedly higher antioxidant capacity. De Santana et al. (2015) [3] reported that this enhanced activity, reflected in both radical scavenging and oxygen radical absorption capacities, is associated with the elevated levels of tocopherol isomers and total phenolic compounds in *P. setacea* samples. Overall, the composition of OPS has been linked to diverse biological effects, including anti-inflammatory, antioxidant, and skin-regenerative activities, underscoring its potential for dermatological applications [4].

Despite these promising attributes, the direct use of natural oils in topical applications is often limited by their undesirable physicochemical and organoleptic properties. These include (i) unfavorable sensorial characteristics (e.g., greasy texture, poor spreadability), (ii) strong odor, and (iii) susceptibility to oxidative degradation, which can negatively impact patient compliance and therapeutic efficacy [5].

To address these challenges, nanotechnology-based delivery systems, particularly microemulsions (MEs), offer a promising solution. Microemulsions are thermodynamically stable, isotropic mixtures typically composed of oil, water, surfactants, and co-surfactants [6]. Their spontaneous formation, high solubilization capacity, ability to enhance dermal permeation, and cost-effectiveness make microemulsions attractive carriers compared with other delivery systems [6–7]. Nevertheless, conventional microemulsions typically require high concentrations of surfactants, which may raise safety concerns such as cytotoxicity and skin irritation [8]. Furthermore, the optimization of microemulsion formulations may be time-consuming and costly, and their stability is often sensitive to environmental factors (e.g., pH, salinity, and temperature). These challenges highlight the importance of a comprehensive understanding of the physicochemical properties and interactions of their constituents [7,9].

In this context, the quality by design (QbD) framework provides a systematic and scientifically grounded approach for pharmaceutical formulation development. QbD emphasizes predefined quality objectives, product and process understanding, and risk management [10]. Key elements include the definition of a quality target product profile (QTPP), identification of critical quality attributes (CQAs), and comprehensive risk assessment (RA) [10]. The QTPP establishes the desired characteristics of the final product to ensure safety, efficacy, and patient acceptability, whereas the CQAs comprise physical, chemical, biological, and microbiological characteristics that must remain within appropriate limits to guarantee product quality [11]. In turn, RA tools complement this framework by identifying potential sources of variability and supporting the systematic optimization of the formulation [10,12].

Therefore, the present study aimed to develop and optimize a low-surfactant microemulsion containing PEG-30 castor oil and Span^®^ 80, based on the approach described by Dourado et al. (2022) [13], as a topical delivery system for *Passiflora setacea* seed oil. QbD principles and experimental design methodologies were employed to guide formulation development. The microemulsion was designed to enhance physicochemical stability, improve skin application performance, and support future therapeutic applications.

## Results and Discussion

### Oil extraction and characterization

OPS was extracted using the Soxhlet method with *n*-hexane as the solvent, yielding 30.5 ± 0.8% (w/w) relative to the initial seed mass. After extraction, the oil was dried using a rotary evaporator to remove residual solvent, filtered through a PTFE membrane (0.45 µm), and stored under refrigerated conditions (4 ± 2 °C) in amber glass bottles to preserve its quality and minimize oxidative degradation and microbiological contamination.

The extraction yield aligns with values reported in the literature for the same species, which range from ≈32% with ethyl ether to ≈34% with hexane [3–5]. Such variations are commonly influenced by parameters including solvent type, moisture content of the seeds, extraction time, and temperature conditions [6].

The chemical profile of OPS was determined after transesterification to fatty acid methyl esters (FAME), followed by gas chromatography coupled with mass spectroscopy (GC–MS) analysis. The relative composition of fatty acids is presented in Table 1, and the corresponding chromatogram is provided in Figure S1 (Supporting Information File 1).

**Table 1:** Chemical composition of fatty acid methyl esters (FAMEs) derived from *Passiflora setacea* seed oil (OPS) as determined by GC–MS.

Compound	Retention time (min)	Composition (%)	Similarity index

impurity	24.17	0.07	–
isopropyl myristate	31.15	0.06	852
palmitic acid	33.17	9.32	967
isopropyl 14-methylpentadecanoate	35.06	1.66	881
linoleic acid	36.42	64.69	968
oleic acid	36.50	20.67	954
10-octadecenoic acid	36.57	0.11	910
stearic acid	36.93	3.36	957
isoarachidic acid	40.38	0.05	801

The major constituents identified in OPS were linoleic acid (64.69%), oleic acid (20.67%), palmitic acid (9.32%), and stearic acid (3.36%). Additional compounds, including isopropyl myristate, isopropyl 14-methylpentadecanoate, 10-octadecenoic acid, and isoarachidic acid, were also detected and are reported here for the first time in this *Passiflora* species. It is important to note that the transesterification process used for GC–MS analysis may yield different proportions of free fatty acids, triglycerides, and transesterified *N*-acyl lipids, potentially affecting the observed lipid profile [14].

The four predominant fatty acids in OPS (i.e., oleic, palmitic, stearic, and linoleic acids) are physiologically relevant to skin health. Oleic, palmitic, and stearic acids are the major components of the epidermal and dermal lipid matrix [15]. The balance among these fatty acids is associated with skin homeostasis, influencing the activity of enzymes that regulate keratinocyte and fibroblast proliferation [15–16]. Furthermore, linoleic acid and oleic acid serve as precursors to various lipid mediators, including prostaglandins, leukotrienes, and lipoxins, which are essential for modulating inflammatory responses and coordinating immune cell activity during wound healing [17–19].

**Figure 1:**
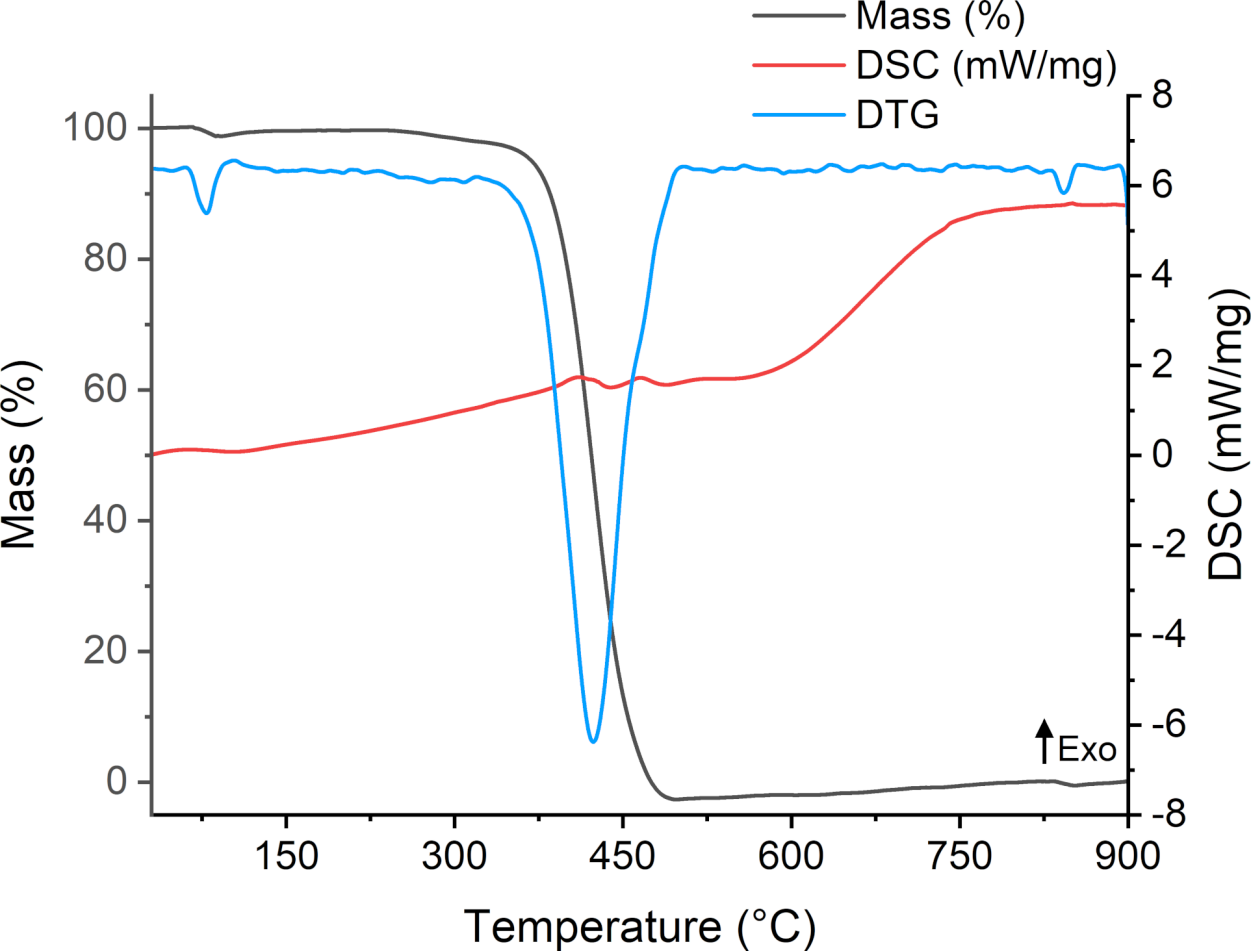
Thermogravimetric analysis, derivative thermogravimetry (DTG), and differential scanning calorimetry (DSC) of *P. setacea* seed oil. Legend: The black line indicates the TGA curve (% mass loss); the blue line corresponds to the DTG; and the red line shows the DSC signal (mW/mg).

In addition to its chemical characterization, the thermal stability in inert atmosphere (N_2_) of OPS was assessed. Thermogravimetric analysis (TGA) revealed an initial mass loss of less than 1% (*T*_onset_ = 60 °C), likely associated with the evaporation of residual solvent entrapped in the oil matrix. A second thermal event began (*T*_onset_ = 384 °C) corresponding to the thermal degradation of the oil (Figure 1). This thermal behavior is consistent with that reported for *P. edulis* seed oil, which exhibited a similar decomposition profile [20].

### Quality by design approach

#### Defining the product profile and critical quality attributes

In this study, the QTPP and associated CQAs were established during the pre-formulation phase, guiding the formulation strategy (Table 2) [21].

**Table 2:** Quality target product profile and corresponding justification for the microemulsion system.

QTPP	Target	Justification

dosage form	microemulsion and gelled microemulsion	thermodynamically stable systems with high permeability and biodistribution suitable for topical delivery
route of administration	topical	appropriate for skin conditions; minimizes systemic toxicity due to limited absorption and distribution
delivery type	modified-release topical system	enables rapid permeation with minimal systemic absorption, enhancing local therapeutic effect
appearance	clear, yellowish, homogeneous	ensures aesthetic appeal and uniformity, reflecting formulation quality
active pharmaceutical ingredient (API) strength	higher oil content	vegetable oil-based API is well tolerated and may enhance therapeutic outcomes
shelf stability	minimum of two months at room temperature (25 ± 2 °C)	maintains formulation integrity and therapeutic potential during preliminary storage period
safety	skin-compatible	formulation must preserve skin barrier functions, including pH and cytocompatibility
efficacy	enhanced local bioavailability	nanostructures improve drug release kinetics and skin permeation

Based on the QTPP, preformulation-level CQAs were systematically identified by assessing how their potential directly or indirectly influences the performance and quality of the final products (Table 3). These attributes encompass physicochemical, biological, and microbiological properties that must remain within defined limits to ensure the desired quality, safety, and efficacy of the microemulsion system [11].

**Table 3:** Preformulation-level CQAs and their justification for inclusion.

Quality attribute	Target value	CQA?	Justification

hydrodynamic diameter	<100 nm	yes	microemulsions are characterized by droplet sizes below 100 nm, which directly impacts system definition and performance
polydispersity index (PdI)	<0.2	yes	a narrow size distribution (low PdI) indicates uniform droplet size and improved stability, reducing risks like Ostwald ripening
pH value	neutral to slightly acidic	yes	ensures compatibility with skin physiology and prevents irritation; pH shifts may signal degradation or instability
viscosity	>10^3^ cP	yes	sufficient viscosity promotes appropriate spreadability, skin adherence, and user acceptability for topical formulations
appearance	transparent	yes	transparency reflects nanoscale droplet size and formulation homogeneity
surface tension	ultralow	yes	low surface tension facilitates spontaneous formation of microemulsions and affects drug solubilization and interfacial behavior
physicochemical stability	thermodynamic, oxidative, and microbiological stability	yes	ensures shelf life, safety, and preservation of the therapeutic properties of a product throughout its use

**Figure 2:**
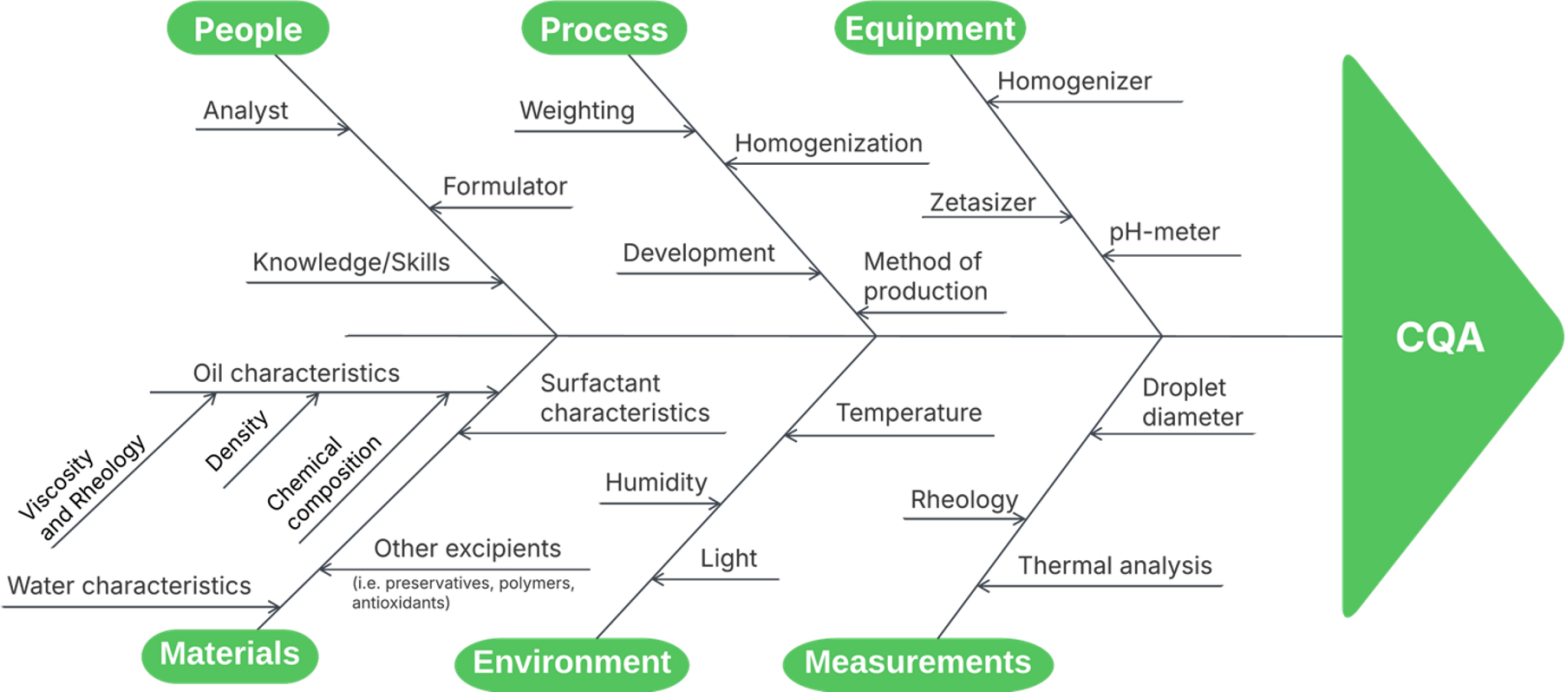
Ishikawa (fishbone) diagram illustrating potential sources of variability affecting CQAs in the development of the microemulsion system. Key influencing categories include people, process, equipment, materials, environment, and measurements, each contributing to formulation performance and quality consistency.

To further assess potential formulation and process risks, an Ishikawa (fishbone) diagram was constructed to visualize and categorize possible sources of variability affecting the identified CQAs (Figure 2) [22]. Following this qualitative analysis, a risk estimation matrix (REM) was employed to quantitatively evaluate these risks. A three-level interdependence scale (1: low, 3: medium, 9: high) was applied to assess the relationship between the QTPP and CQAs (Supporting Information File 1, Table S1), as well as between the CQAs and the critical material attributes (CMAs) and critical process parameters (CPPs, Supporting Information File 1, Tables S2 and S3) [23].

**Figure 3:**
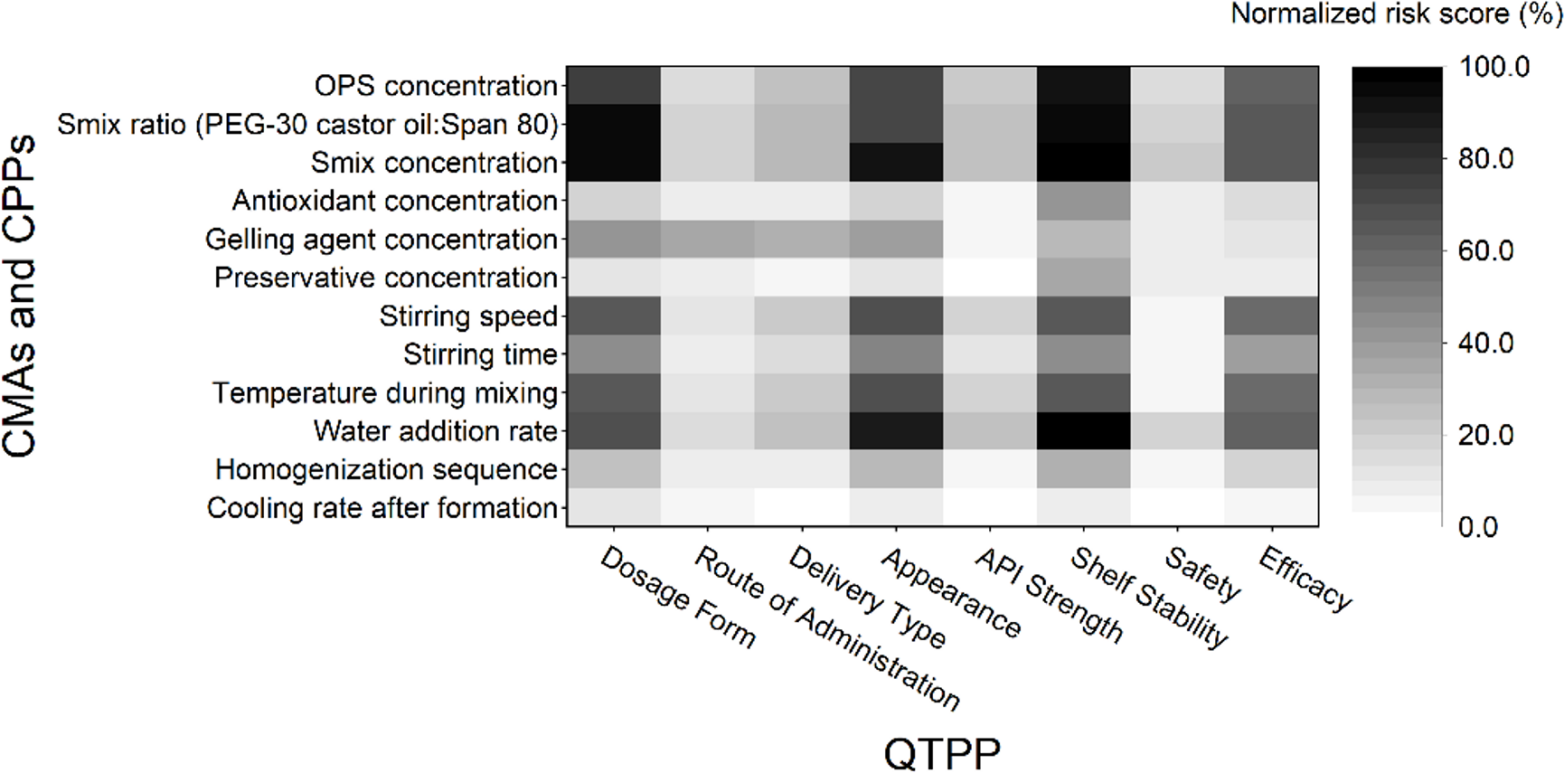
Heatmap of the normalized risk scores (%) illustrating the impact of CMAs and CPPs on each element of the QTPP. Higher risk scores (darker shades) indicate stronger influence on final product quality and guide prioritization in formulation control strategies. OPS concentration, *S*_mix_ ratio (PEG-30 castor oil/Span^®^ 80), and *S*_mix_ concentration emerged as key high-risk variables.

To quantify the overall influence of each CMA and CPP on the final product quality, the two-risk estimation matrices (QTPP–CQA and CQA–CMA/CPP) were mathematically combined. The resulting composite scores are presented as a heatmap in Figure 3, where darker regions indicate variables with a higher potential to compromise the ability of the product to meet the predefined QTPP. This risk prioritization informed the selection of input variables for the design of experiments (DoE) and future control strategies. Specifically, OPS concentration, surfactant mixture (*S*_mix_) ratio (PEG-30 castor oil/Span^®^ 80), and *S*_mix_ concentration were identified as high-risk factors and selected for the DoE due to their significant impact on the CQAs. Other high-risk variables, while not included in the DoE, were flagged for strict monitoring during formulation to ensure quality consistency.

### Experimental design for the development and optimization of *Passiflora setacea* microemulsion

An initial exploratory 2^3^ full factorial applied was employed to evaluate the effects and interactions among key formulation variables (see Supporting Information File 1, Tables S4 to S6). However, the presence of significant higher-order interactions limited the predictive capability and overall fit of the model. These limitations indicated the need for a more refined strategy with higher resolution and reduced confounding effects. Therefore, a Box–Behnken design (BBD) was selected for the subsequent optimization phase [24].

The BBD employs three levels for each factor: in each experiment, two factors vary between their upper (+1) and lower (−1) levels, while the third remains at the center point (0) [24]. Using this approach, 17 experimental combinations were generated to systematically explore the formulation space. The specific combinations and corresponding response results for all experimental runs are summarized in Table 4.

**Table 4:** Experimental execution and responses from the Box–Behnken design^a^.

Standard order	Execution order	Factor A (%)	Factor B (mass ratio)	Factor C (%)	Response 1 (nm)	Response 2 (PdI)	Response 3 (visual class)

1	9	5	7:3	15	23.7	0.10	2
2	6	15	7:3	15	59.9	0.21	3
3	15	5	9:1	15	20.3	0.07	1
4	13	15	9:1	15	246.2	0.32	4
5	11	5	8:2	10	27.4	0.10	2
6	8	15	8:2	10	201.2	0.16	4
7	2	5	8:2	20	19.6	0.05	1
8	3	15	8:2	20	45.9	0.19	3
9	17	10	7:3	10	47.0	0.18	3
10	7	10	9:1	10	182.7	0.30	4
11	16	10	7:3	20	24.0	0.16	2
12	12	10	9:1	20	30.1	0.14	2
13	4	10	8:2	15	38.5	0.16	3
14	1	10	8:2	15	41.0	0.14	3
15	10	10	8:2	15	36.4	0.12	3
16	5	10	8:2	15	37.1	0.20	3
17	14	10	8:2	15	33.4	0.18	3

**^a^**Factor A: oil concentration (*P. setacea*); Factor B: surfactant mixture ratio (PEG 30 castor oil:Span^®^ 80); Factor C: surfactant mixture concentration. Response 1: hydrodynamic diameter (nm); Response 2: polydispersity index; Response 3: visual classification (1–clear to 4–turbid).

**Figure 4:**
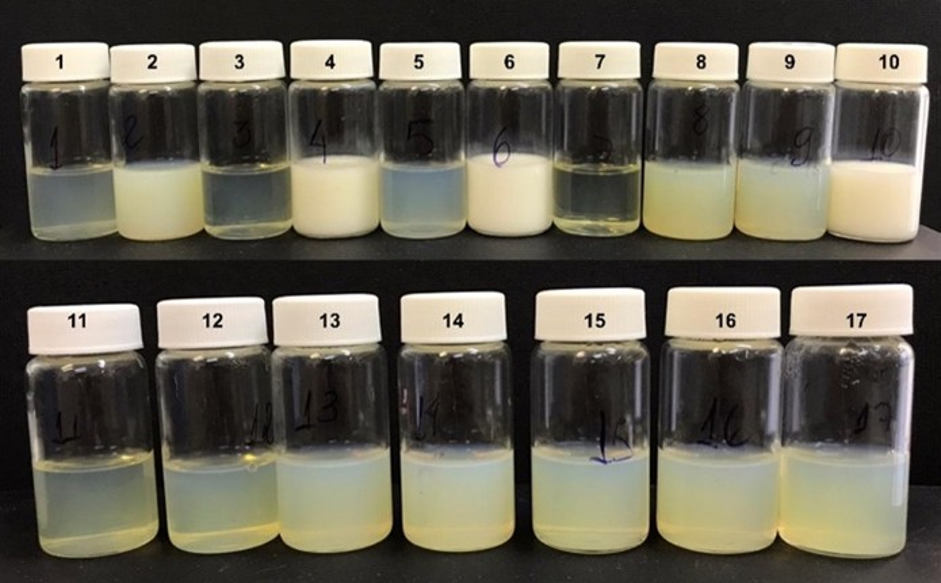
Microemulsion systems obtained from Box–Behnken design experimental runs. Each formulation is labeled according to its standard order in the design matrix.

Although the BBD does not include extreme combinations of variables, it improved precision and accuracy for predictions near the center of the experimental space. The prepared formulations corresponding to the experimental design runs are shown in Figure 4, and a summary of the BBD is presented in Table 5.

**Table 5:** Summary of Box–Behnken design responses and model fitting^a^.

Response	Min.	Max.	Avg.	SD	Ratio	Transformation	Model type

Dh (nm)	19.6	246.2	65.6	70.7	12.54	√ (Dh)	reduced cubic
PdI	0.05	0.32	0.16	0.07	6.59	1/√ (PdI)	reduced quadratic
visual score	1	4	2.71	0.99	4.00	none	reduced quadratic

^a^Abbreviations: Dh = hydrodynamic diameter; PdI = polydispersity index; SD = standard deviation; Ratio = Max/Min; Transformation = Box–Cox transformation applied for model fitting.

To refine the model and improve predictive accuracy, the response data were subjected to Box–Cox transformation, an essential step when non-normal distribution is detected [25]. Following transformation, analysis of variance (ANOVA) was conducted to evaluate the statistical significance of the overall model and the individual formulation factors. To enhance model parsimony, backward regression elimination was applied to remove nonsignificant terms (*p* > 0.1), while preserving model hierarchy where required [26]. A detailed summary of these findings is available in the Supporting Information File 1 (Tables S7 to S9).

**Figure 5:**
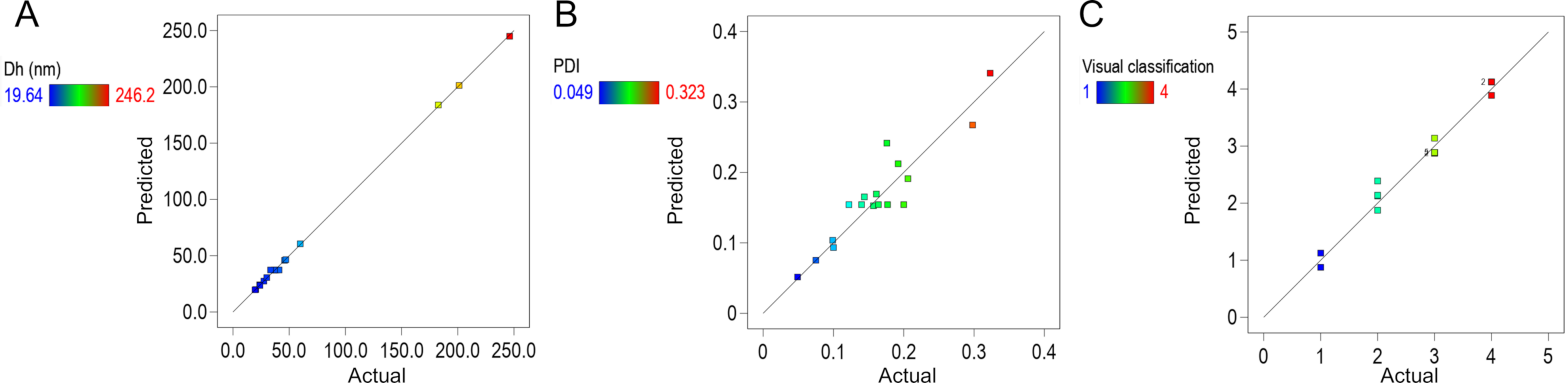
Linear regression plots of actual versus predicted values for: (A) hydrodynamic diameter, (B) polydispersity index, and (C) visual classification. The high degree of correlation between predicted and experimental values supports the robustness and predictive capability of the fitted models.

The final regression models for hydrodynamic diameter, polydispersity index, and visual classification exhibited strong statistical performance. Lack-of-fit tests were nonsignificant for both Dh (*p* = 0.632) and PdI (*p* = 0.665), confirming good model–data agreement. The *R*^2^ values for Dh, PdI, and visual classification were 0.99, 0.94, and 0.97, respectively, with adjusted *R*^2^ values of 0.99, 0.89, and 0.95. These metrics indicate that the models account for a high proportion of the variability in the measured responses (Figure 5).

These findings confirm that the selected formulation variables had a statistically significant and predictable impact on key microemulsion attributes. The strength of the fitted models supports their use in optimizing the formulation through the DoE approach. The predictive models for hydrodynamic diameter, polydispersity index, and visual classification were expressed as a polynomial function of the encoded factor as follows:


[1] $$\begin{array}{c} \sqrt{\text{Dh}}=6.10+3.51\text{A}+1.85\text{B}-2.50\text{C}+2.08\text{AB}-1.65\text{AC}\\ -1.52\text{BC}+1.03{\text{A}}^{2}+1.06{\text{B}}^{2}+0.52{\text{C}}^{2}+0.44{\text{A}}^{2}\text{C}\\ -0.69{\text{AC}}^{2}, \end{array}$$

[2] $$\begin{array}{c} \frac{1}{\sqrt{\text{PdI}}}=2.57-0.72\text{A}-0.05\text{B}+0.26\text{C}-0.23\text{AB}\\ -0.39\text{AC}+0.48{\text{A}}^{2}-0.30{\text{B}}^{2}, \end{array}$$

[3] $$\begin{array}{c} \text{Visual classification}=2.89+1.00\text{A}+0.13\text{B}-0.63\text{C}\\ +0.50\text{AB}-0.25\text{BC}-0.39{\text{A}}^{2}. \end{array}$$

**Figure 6:**
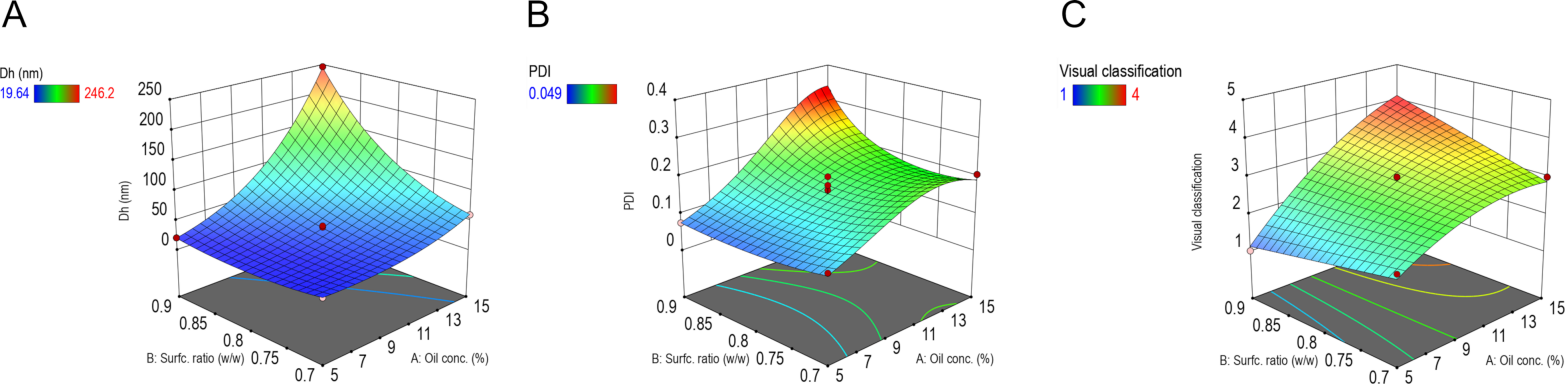
Surface plots derived from the Box–Behnken design showing the effects of formulation variables on: (A) hydrodynamic diameter, (B) polydispersity index, and (C) visual classification. The plots illustrate the interaction between key input variables, with the third variable held constant at its central level.

These equations were then used to generate 3D response surface plots for each response, as shown in Figure 6. The axes were selected based on the most significant interactions identified through ANOVA, while the third factor was held constant at its central level. The resulting plots provide a visual representation of the individual and interactive effects of the formulation variables. As expected, increasing oil concentration was associated with higher hydrodynamic diameter, PdI, and visual classification scores. In contrast, higher concentrations of the *S*_mix_ led to reductions in all three response values, indicating microemulsion uniformity and clarity.

The objective of this experimental design was to develop a microemulsion that meets CQAs, specifically achieving a smaller hydrodynamic diameter, lower polydispersity index, and improved visual classification. Additionally, minimizing the concentration of *S*_mix_ was a priority, given the potential cytotoxic effects of high surfactant levels. Based on these criteria, numerical optimization was applied to predict the optimal combination of formulation factors. This approach relies on a mathematical desirability function, in which each factor and response is assigned a value between 0 (unacceptable) and 1 (fully desirable). The relative importance of each goal is then weighted on a scale from 1 to 5, allowing for balanced multiresponse optimization. These predefined parameters are subsequently processed by the software algorithm to identify the optimal combination of responses [27]. The constraints and results of the microemulsion optimization process are summarized in Table 6.

**Table 6:** Constraints and results of numerical optimization for microemulsion development.

Variable/response	Goal	Lower limit	Upper limit	Importance (1–5)	Optimized result

A: oil concentration (%)	in range	5	15	3	5
B: proportion of surfactants (*S*_mix_)	in range	7:3	9:1	3	9:1
C: *S*_mix_ concentration (%)	minimize	10	20	3	15
hydrodynamic diameter (nm)	minimize	19.6	246.2	5	19.9
polydispersity index	minimize	0.05	0.32	5	0.08
visual classification	target = 1	1	4	5	1

The optimized microemulsion system, selected based on predefined criteria, comprised 5% of oil, a 9:1 (w/w) *S*_mix_ ratio of PEG-30 castor oil to Span^®^ 80, and 15% of this *S*_mix_. Microemulsions typically require high surfactant and/or co-surfactant concentrations to sufficiently reduce interfacial tension and promote spontaneous formation of stable nanodroplets [6,28]. However, a previous study from our group (Dourado et al. (2022) [13]) successfully developed stable microemulsion regions using the same surfactant blend at similar concentrations (13.2% of PEG-30 castor oil and 1.8% of Span^®^ 80), for curcumin delivery in a Miglyol^®^ 812N (medium chain triglycerides) oil phase. These findings support the feasibility of developing microemulsions with reduced surfactant content, in alignment with safety considerations and current best practices in nanocarrier formulation [29].

The application of experimental design in microemulsion development is well established, with several studies employing DoE strategies to obtain and optimize microemulsified systems, particularly for topical use [30–34]. The primary advantage of this approach lies in its regulatory relevance within the QbD framework, as it allows the identification of operational factor ranges that ensure compliance with CQAs.

### Gelled microemulsion

For topical administration, the incorporation of adjuvants is necessary to enhance the performance of the formulation. One of the main limitations of microemulsions for this route is their inherently low viscosity, which is undesirable for cutaneous application. Moreover, polyunsaturated fatty acids are prone to oxidative degradation, compromising their biological efficacy. Another frequent issue in emulsified systems is microbial contamination, which can result in physical and chemical instability, such as pH shifts, turbidity, degradation of active components (e.g., fatty acids), and phase separation [35].

To address these challenges and improve topical suitability, additional excipients were incorporated into the formulation within their commonly employed concentration ranges [36]. Sodium carboxymethylcellulose (NaCMC, 1.5% w/w) was added as a gelling agent owing to its excellent biocompatibility, ease of dispersion in aqueous systems, and ability to enhance viscosity without compromising droplet stability [37]. Vitamin E at 0.05% w/w was included as a lipophilic antioxidant to protect the unsaturated fatty acids in OPS oil from oxidative degradation, thereby improving formulation stability [38]. Additionally, 0.2% of Liquid Germall^®^ Plus was selected as a broad-spectrum preservative for the aqueous phase, with regulatory approval for topical applications.

### Characterization of microemulsions and physicochemical stability

#### Physicochemical stability

As prove of the model prediction capabilities, the optimized formulation identified through numerical optimization, the base microemulsion, was produced and characterized to confirm its quality and alignment with the CQAs. On the first day post-production, the formulation exhibited a hydrodynamic diameter of approximately 22.0 nm and a PdI of 0.14, both of which fall within the acceptable CQA criteria.

**Figure 7:**
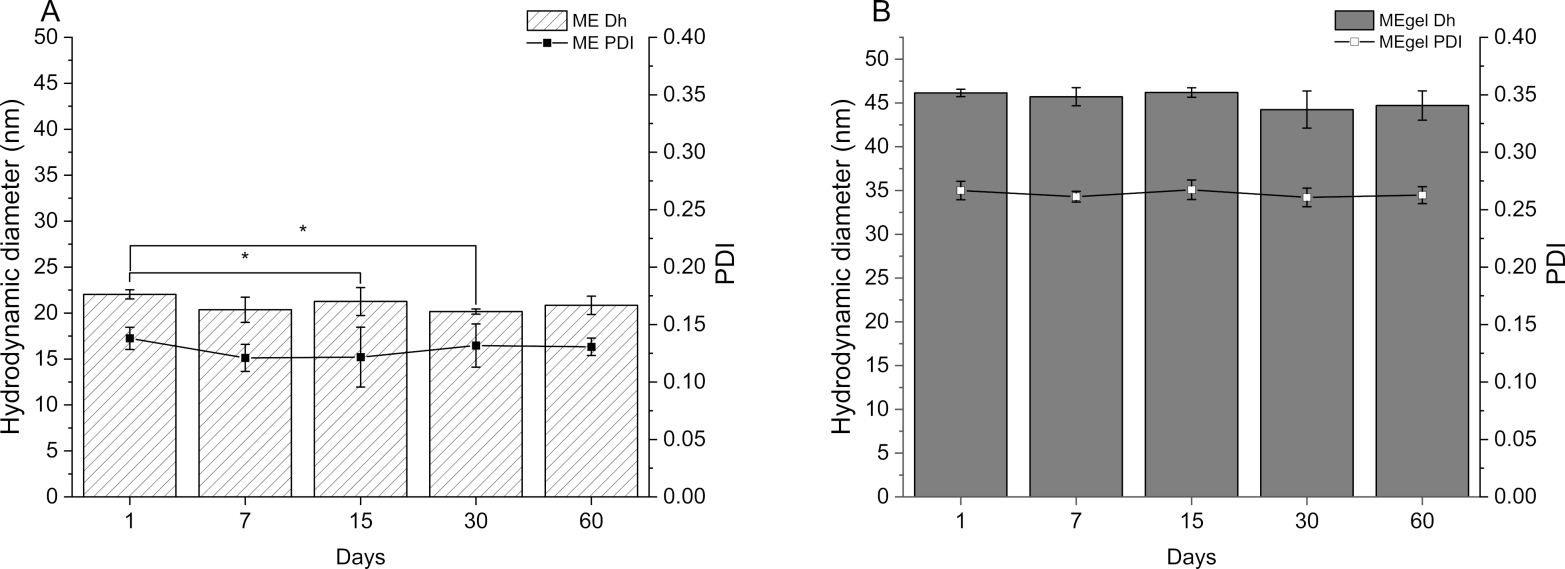
Hydrodynamic diameter over a 60 day period for (A) the optimized microemulsion (ME) and (B) the gelled microemulsion (MEgel). Data are presented as mean ± SD (*n* = 3). *p < 0.05 indicates statistically significant difference.

The stability of the system was evaluated over a 60 day period (Figure 7A). Throughout this time, no significant changes were detected in particle size, with the hydrodynamic diameter consistently remaining below 100 nm, and the PdI remaining below 0.20, confirming the physical stability of the base microemulsion and its preservation of desirable nanostructural features over time [39].

It is important to note, however, that NaCMC forms polymeric networks when dispersed in aqueous media, which scatter light and may artificially increase the measured hydrodynamic diameter [40]. Consequently, dynamic light scattering (DLS) data may not accurately reflect the true droplet size distribution in the MEgel. This phenomenon is evident from the apparent increase in mean hydrodynamic diameter to 45.1 nm and PdI to 0.27, which is attributed to NaCMC matrix scattering rather than actual droplet growth [40]. A comparison of the hydrodynamic diameter distributions for both systems is provided in Figure S2 (Supporting Information File 1). The stability profile of the MEgel formulation is presented in Figure 7B.

**Figure 8:**
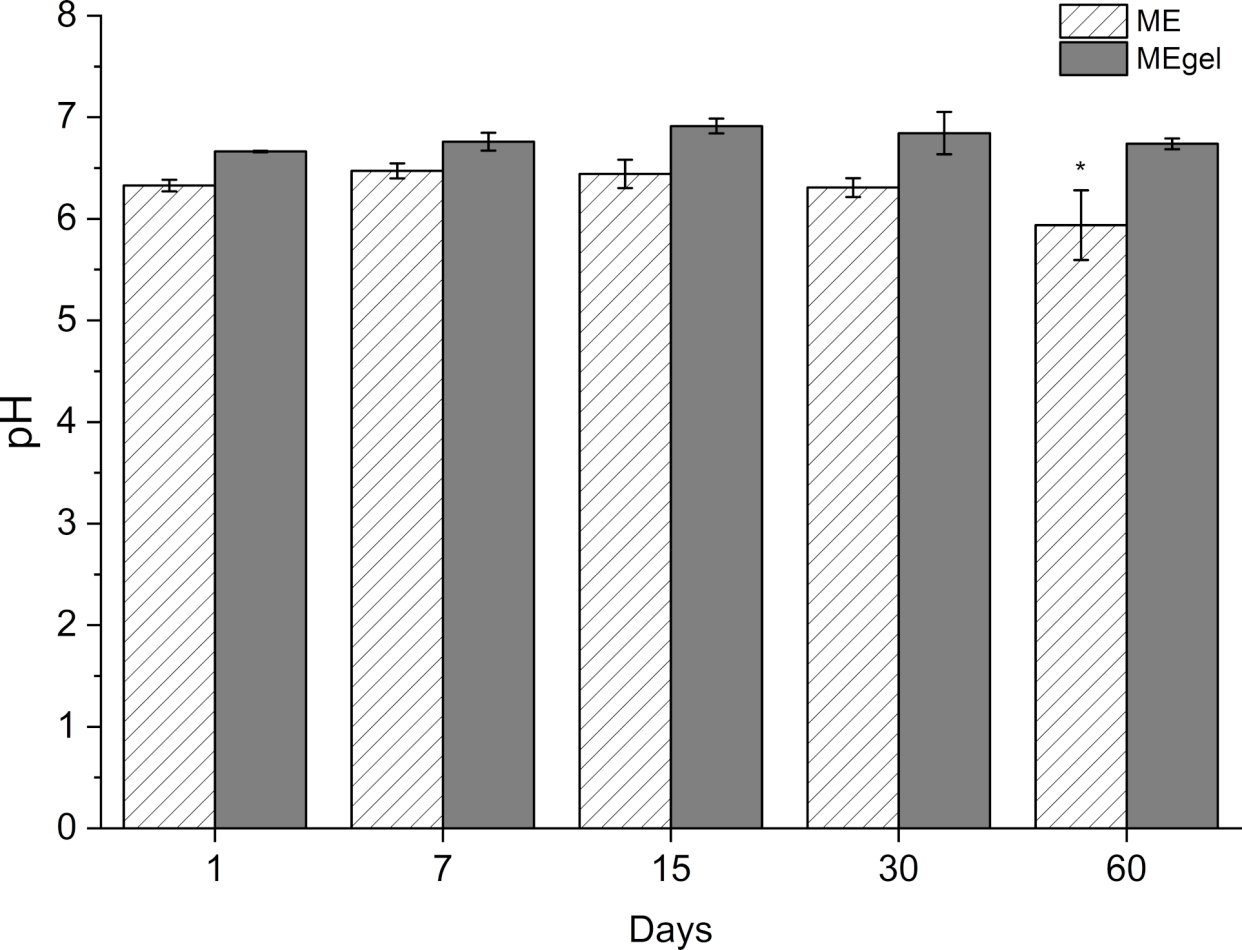
pH values of ME and MEgel formulations over a 60 day storage period. Data are presented as mean ± SD (*n* = 3). *p < 0.05 indicates a statistically significant difference.

Furthermore, as shown in Figure 8, both ME and MEgel formulations maintained stable pH values throughout the storage period, with mean values of 6.3 ± 0.3 and 6.8 ± 0.1, respectively. The slightly acidic pH of healthy skin (typically ranging from 4 to 6) plays a key role in supporting physiological functions such as keratinocyte differentiation, lipid mantle formation of the stratum corneum, and preservation of the skin microbiome. In contrast, compromised or sensitive skin often exhibits a shift toward neutral pH [41]. Given their stability and proximity to the natural skin pH, the observed pH values for ME and MEgel are considered appropriate for topical application.

Nevertheless, on day 60 the ME formulation exhibited a statistically significant difference (*p* < 0.05) in pH compared with earlier time points. Although the reduction was modest, from pH 6.4 ± 0.1 to 5.9 ± 0.3, it is hypothesized to result from a combination of physicochemical and microbiological factors [42]. One possible explanation involves the oxidative or hydrolytic degradation of the oil phase, particularly the fatty acids in OPS, as well as the surfactants. In particular, the autoxidation of the polyoxyethylene (POE) chains in the hydrophilic head group of the PEG-30 castor oil and the hydrolysis of the surfactant hydrophobic tails may have contributed to the observed pH shift [42–43]. These findings underscore the importance of incorporating preservatives into the MEgel formulation to mitigate such instability.

It is worth noting that although microemulsified systems are thermodynamically stable, their individual excipients remain vulnerable to instability phenomena such as chemical degradation and microbial contamination. These processes can compromise the thermodynamic stability and overall performance of the system [6].

#### Thermal analysis

**Figure 9:**
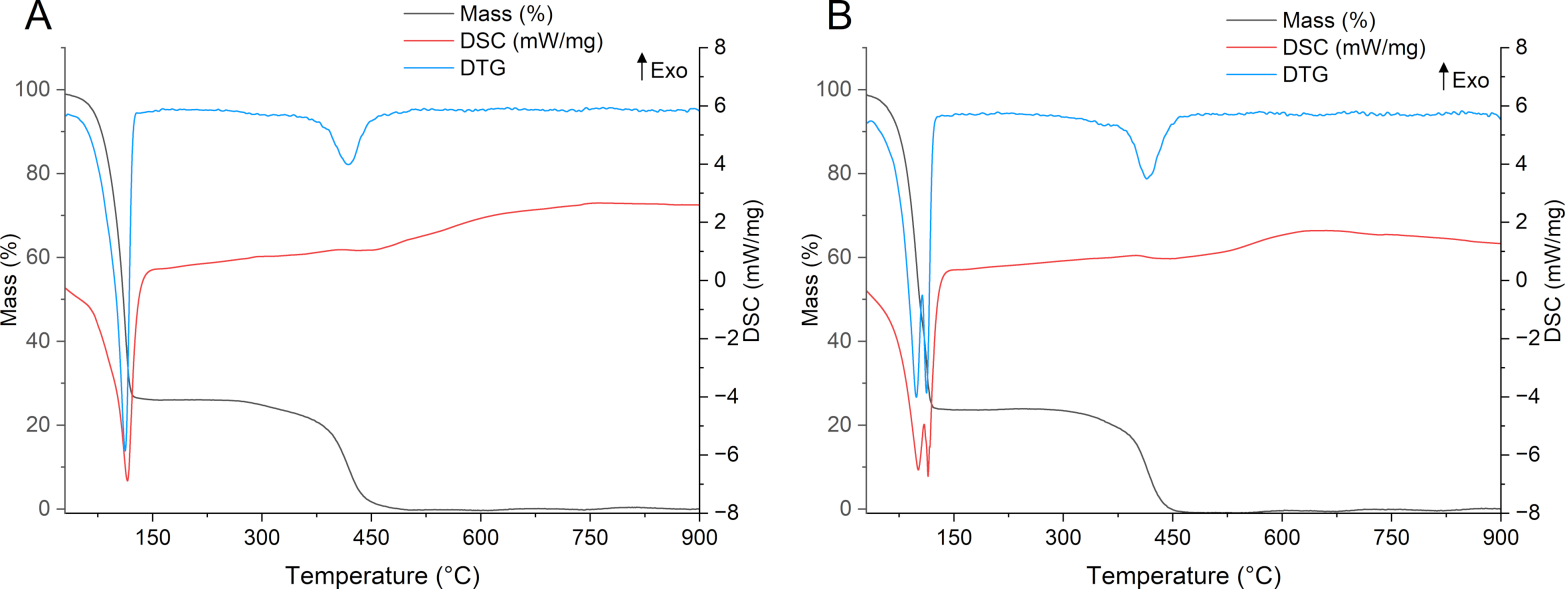
Thermal analysis of ME and MEgel. (A) Differential thermal and thermogravimetric profiles of the base microemulsion. (B) Differential thermal and thermogravimetric profiles of the gelled microemulsion.

The thermal characteristics of the formulations were also evaluated (Figure 9). The thermograms revealed no evidence of incompatibility among the excipients, such as the lowering of degradation temperatures. The thermal events observed in the DTG and DSC curves correspond to the loss of free water (≈98 °C), solvation water (≈110 °C), and thermal degradation of the oil phase (≈417 °C), overlapping with the degradation profiles of other components such as NaCMC and vitamin E [44–45].

#### Surface tension

The surface tension of the microemulsion was characterized through six replicates, yielding an average value of 40.27 ± 0.22 mN·m^−1^. Similar surface tension values have been reported for microemulsions in the literature [46–47]. This range is considerably favorable for topical formulations, as lower surface tension enhances spreadability and promotes interaction with the stratum corneum and its lipidic barrier. Due to instrumental limitations associated with the high viscosity resulting from NaCMC addition, the surface tension of the MEgel could not be determined. However, NaCMC solutions exhibit negligible surface activity and typically maintain surface tension values close to that of water [48].

#### Rheological analysis

**Figure 10:**
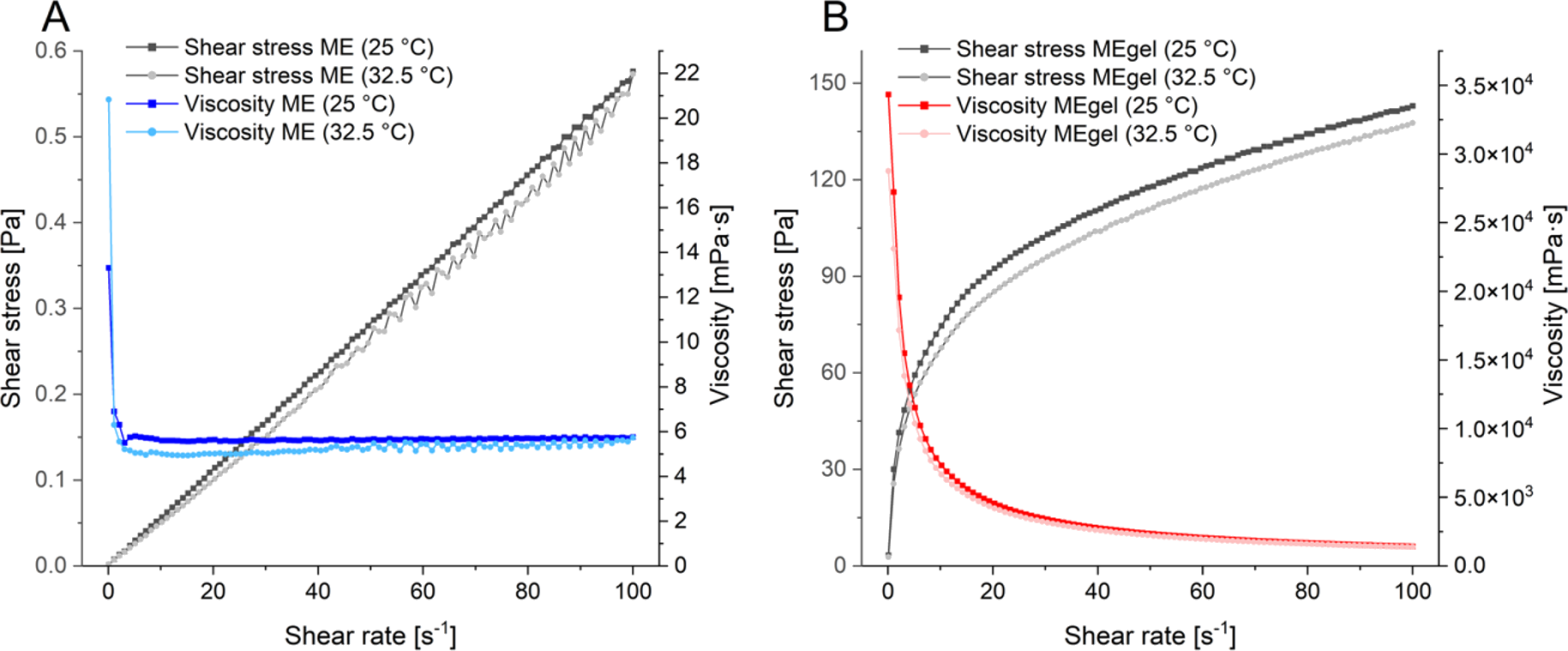
Shear stress and viscosity profiles of (A) the base microemulsion, and (B) the gelled microemulsion at 25 and 32.5 °C.

Rheological profiling provides key insights into the behavior of formulations under applied stress or strain, offering critical information about viscosity, flow characteristics, and structural stability. According to the proposed QTPP, a higher viscosity with pseudoplastic (shear-thinning) behavior is preferred for topical formulations, as it ensures ease application and good retention on the skin. Accordingly, the microemulsions were evaluated for their shear stress and viscosity at both room temperature (25 ± 2 °C) and skin temperature (32.5 ± 2 °C). Microemulsions can exhibit diverse rheological behaviors depending on their type, structure, droplet density, and interdroplet interactions [49]. As shown in Figure 10A, the base ME demonstrated Newtonian behavior, with viscosity remaining constant regardless of shear rate or temperature. This is consistent with microemulsions that possess globular or discontinuous droplet structures, which typically exhibit Newtonian flow characteristics [50–51].

However, MEgel exhibited a pseudoplastic, shear-thinning behavior, as demonstrated by a decrease in viscosity from approximately 3.0 × 10^4^ to 1.4 × 10^3^ mPa•s. with increasing shear rate (Figure 10B). This rheological profile enhances the residence time of the formulation on the skin and minimizes runoff, thereby improving patient compliance.

### In vitro cytocompatibility

**Figure 11:**
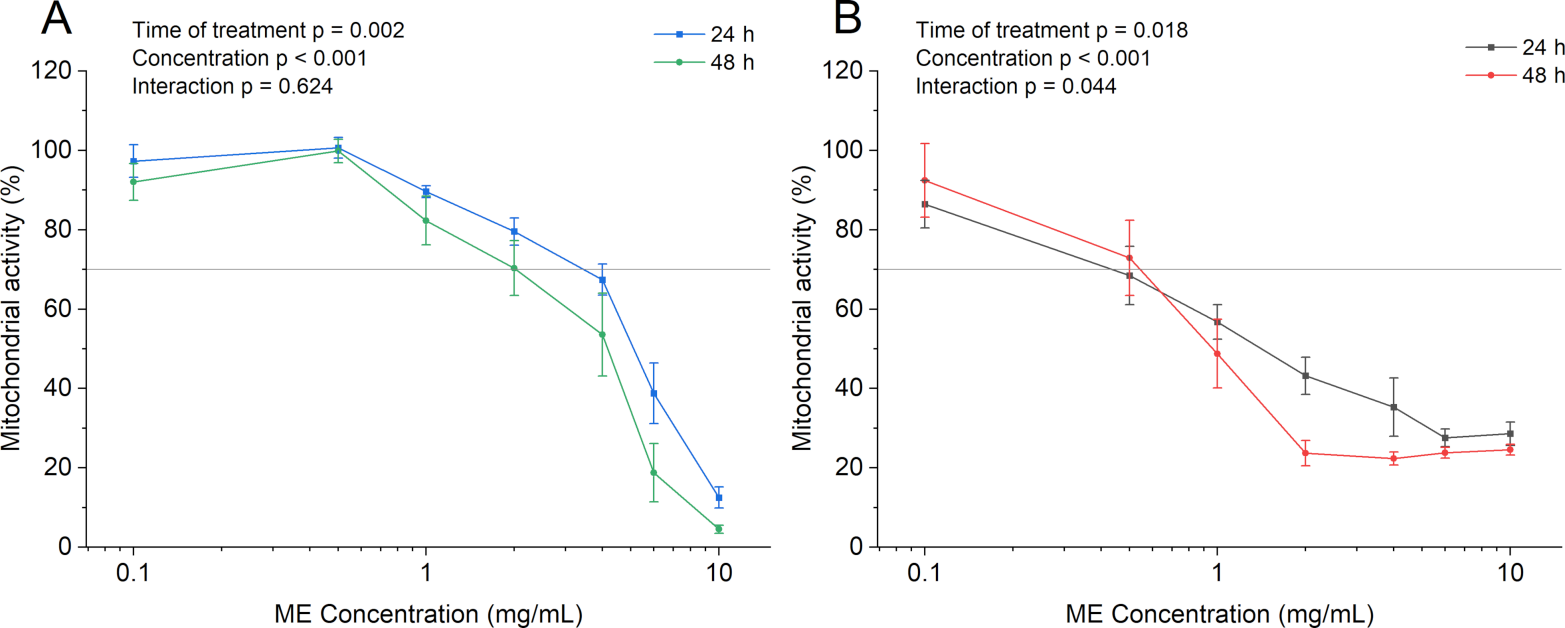
Mitochondrial activity of (A) RAW 264.7 murine macrophages and (B) HUVECs after 24 h and 48 h of exposure to different concentrations of the microemulsion, assessed by the MTT assay. Data are presented as mean ± SD (*n* = 3).

To assess the safety of the ME formulation for potential topical use, a proof-of-concept cytocompatibility evaluation was conducted using the MTT assay, which measures mitochondrial metabolic activity. The assay was performed on two cell lines relevant to wound healing and topical application: murine macrophages (RAW 264.7) and human umbilical vein endothelial cells (HUVECs) (Figure 11). According to ISO 10993-13 guidelines, the base ME was considered cytocompatible up to concentrations of 2 mg/mL for RAW cells and 0.5 mg/mL for HUVECs. The observed cytotoxicity at higher concentrations is likely due to the increase proportion of surfactants in the formulation. Additionally, under certain experimental conditions, fatty acids may induce lipid peroxidation and trigger pro-inflammatory responses in endothelial cells such as HUVECs, potentially offsetting the proliferative effects typically observed in keratinocytes such as HaCaT cells [2,52–53].

To gain a more comprehensive understanding of the therapeutic potential of OPS and its ME formulation in wound healing and topical applications, it is crucial to extend cytocompatibility assessments to include additional cell types, particularly keratinocytes and dermal fibroblasts, which are key players in skin regeneration and re-epithelialization [17]. Although the MTT assay is a widely accepted and convenient method for preliminary cytotoxicity screening, it offers limited insight into the complex biological processes involved in wound healing. Specifically, it does not evaluate critical mechanisms such as cell migration, proliferation, extracellular matrix remodeling, or the modulation of inflammatory mediators. Therefore, future investigations should incorporate complementary assays that capture these dimensions. Ultimately, in vivo models remain the gold standard for assessing wound healing efficacy, as they replicate the dynamic, multicellular, and inflammatory nature of living tissue, providing crucial translational insights that cannot be obtained through in vitro testing alone [54].

## Conclusion

*Passiflora setacea* seed oil was successfully extracted using the Soxhlet method, yielding 30.5 ± 0.8% (w/w). The oil is rich in polyunsaturated fatty acids, primarily ω-6 (64.7%) and ω-9 (20.7%), which are associated with anti-inflammatory, antioxidant, and regenerative properties, key attributes for dermatological applications. For topical administration, the base microemulsion was supplemented with vitamin E and Liquid Germall^®^ Plus and subsequently gelled with NaCMC, resulting in a pseudoplastic system with suitable viscosity for skin application. In cytocompatibility assays, the ME preserved mitochondrial activity at concentrations up to 0.5 mg/mL in HUVEC and 2 mg/mL in RAW 264.7 cells after 48 h. Toxicity observed at higher concentrations was attributed to increased surfactant content, highlighting the importance of formulation optimization. Despite these promising results, further studies are required to address the limitations identified, including droplet size characterization of MEgel, comprehensive cytotoxicity assessments, and wound healing assays. Overall, the developed microemulsion system represents a promising nanocarrier-based strategy for topical delivery, effectively combining the functional benefits of *P. setacea* seed oil with a QbD-guided formulation process.

## Experimental

### Materials

Seeds of *Passiflora setacea* BRS Pérola do Cerrado were kindly provided by EMBRAPA (Brasília, DF, Brazil – SisGen Registration Nº. A51F883). *n-*Hexane was purchased from LabSynth Ltda. (Diadema, Brazil), and HPLC grade *n-*heptane (Neon Ltda. – Suzano, Brazil) was donated by the Agricultural School of Jundiaí (EAJ-UFRN). The following excipients were donations: Vitamin E (Galena – Campinas, Brazil), Liquid Germall^®^ plus (Mapric – São Paulo, SP), ALKEST^®^ CSO 300 (polyethoxylated castor oil 30 mol, “PEG 30”, Oxiteno – São Paulo, SP) and sodium carboxymethyl cellulose (NaCMC – All chemistry, São Paulo, Brazil). Span^®^ 80 was obtained from Sigma-Aldrich Inc. (St. Louis, MO – USA).

### Extraction of *Passiflora setacea* oil

Oil extraction was performed using a Soxhlet apparatus with 4:1 ratio of *n-*hexane to *P. setacea* seeds, following the methodology recommended by the Association of Official Analytical Chemists [55]. Prior to extraction, dried seeds were grounded, placed in folded filter paper, and inserted into the Soxhlet extractor. The extraction was carried out for 6 h at 60 °C. Afterward, the solvent was removed using a rotary evaporator under reduced pressure at a water bath temperature of 40 °C. The obtained oil was then filtered through a 0.45 µm PTFE membrane and stored in amber glass bottles under refrigeration (4 °C) until use. The oil yield was calculated using the following equation:


[4] $$\text{Yield}\left(\%\;\text{w}/\text{w}\right)=\left(\frac{\text{weight of oil}}{\text{weight of dried seeds}}\right)\times 100$$

### Characterization of *Passiflora setacea* oil

#### Oil composition

The chemical composition of *Passiflora setacea* oil was determined through the analysis of its fatty acids methyl esters (FAMEs) [14,56]. Briefly, 10 mg of oil was solubilized in 1 mL of freshly prepared 0.5 M methanolic potassium hydroxide. The base-catalyzed transesterification reaction was carried out at 60 °C for 15 min. After cooling, the reaction was quenched with 20 µL of acetic acid. To extract the FAMEs, 1 mL of *n*-hexane and water were added, followed by vigorous agitation. Once phase separation occurred, the organic (*n*-hexane) layer was collected and washed three times with saturated NaCl solution to remove residual reagents. A 200 µL aliquot of the organic phase was then dried at room temperature in pre-weighted vials.

FAME quantification was performed using a gas chromatograph (GC Agilent 8860, Agilent – Santa Clara, USA) coupled with a mass spectrometer (5977B GC/MSD, Agilent – Santa Clara, USA). Prior to injection, samples were dissolved in 1 mL of *n-*heptane. The GC system operated with helium as the carrier gas at a flow rate of 1 mL·min^−1^. The injection and detection temperatures were set at 270 °C and 250 °C, respectively. The oven temperature program ranged from 70 °C to 300 °C, increasing at a rate of 3 °C·min^−1^. Compound identification was conducted by matching the mass spectra to those in the NIST17.

#### Simultaneous thermal analysis

The thermal behavior of *P. setacea* oil was evaluated using a simultaneous thermal analysis (STA 449 F3 Jupiter^®^, NETZSCH – Selb, Germany). Samples were weighed in alumina crucibles and analyzed with a heating rate of 10 °C·min^−1^, under nitrogen flow of 10 mL·min^−1^, over a temperature range of 25 °C to 900 °C. Microemulsion systems were analyzed under the same conditions for comparative purposes.

### Quality by design framework

The development of the microemulsion system was guided by a QbD strategy, aligned with ICH Q8(R2) guidelines [57]. This framework was employed to define the QTPP and CQAs, CMAs, and CPPs relevant to the topical delivery of *P. setacea* seed oil.

A risk assessment was conducted to identify and prioritize factors most likely to affect the CQAs. This included the construction of an Ishikawa diagram followed by a REM [58]. The REM was based on interdependence scores between QTPP-CQA and CQA-CMA/CPP relationships, using a three-level scale (1 = low, 3 = moderate, 9 = high). The multiplication of the matrices resulted in a final risk score linking each QTPP element to the corresponding CMA and CPPs, enabling the identification of high-risk variables for subsequent optimization steps.

#### Experimental design for development and optimization of *Passiflora setacea* microemulsion

Experimental design is a valuable strategy for minimizing the number of experiments while maximizing the understand of how each factor influences a dependent variable [59]. Based on variables identified during risk assessment, DoE was applied to investigate the effects of OPS concentration, the surfactants mixture ratio (*S*_mix_), and *S*_mix_ concentration on the formation of a microemulsified system with desirable hydrodynamic diameter and transparency (Table 7).

**Table 7:** Independent and dependent variables used in the experimental planning for the development of *P. setacea* oil-based microemulsions.

Variable	Level (coded)		

Independent variable	lower (−1)	central (0)	upper (+1)

X1: OPS concentration	5%	10%	15%
X2: surfactant ratio (PEG 30 Castor Oil/Span^®^ 80)	7:3	8:2	9:1
X3: *S*_mix_ concentration	10%	15%	20%

Dependent variables	Desired outcomes		

Y1: hydrodynamic diameter (nm)	less than 100 nm		
Y2: polydispersity index	less than 0.2		
Y3: visual classification^a^	category 1 (transparent)		

^a^Y3 was used only on the Box–Behnken design.

Initially, a full factorial design (2^3^) with three central points was employed to explore the experimental domain. Subsequently, a three-level Box–Behnken design was used to refine the model and enhance resolution. Microemulsion clarity was visually classified into four categories: (1) transparent, (2) slightly cloudy, (3) slightly milky, and (4) milky. The experimental matrix and statistical analyses were conducted using Design-Expert^®^ software (M/s Stat-Ease Inc.).

### Production method for *Passiflora setacea* oil-based systems

The formulations were prepared using the phase inversion composition method [60]. Component proportions were defined according to the experimental matrix (Table 7). Briefly, accurately weighed amounts of OPS and surfactants were homogenized under magnetic stirring for 2 min. Ultrapure water was then added dropwise (≈0.5 mL/min) under continuous stirring (≈900 rpm, IKA^®^ C-MAG HS7 – Staufen, Germany). The entire process was conducted at 75 ± 3 °C. After water addition, the mixture was stirred for an additional 15 min to ensure system stabilization.

For the preparation of the topical microemulsion, the same method was followed with slight modifications. Initially, vitamin E (0.05% w/w) was solubilized in the oil phase. The microemulsion was then prepared as previously described. Subsequently, Liquid Germall^®^ Plus (0.2% w/w) and sodium carboxymethylcellulose (1.5% w/w) were added under mechanical stirring to obtain the gelled topical microemulsion.

### Characterization of the emulsified systems

#### Hydrodynamic diameter distribution

The hydrodynamic diameter and polydispersity index of the microemulsions were determined by dynamic light scattering using a Zetasizer^®^ Nano ZS (Malvern Panalytical Ltd. – Malvern, United Kingdom) at 25 °C with a fixed backscattering angle of 173°. Prior to analysis, samples were diluted in purified water (1:40 v/v) [13]. All measurements were performed in triplicate.

#### Surface tension

Surface tension was measured using the pendant droplet method with a drop-shape analyzer (DAS-100, KRÜSS GmbH, Hamburg, Germany). Microemulsion droplets (17 µL) were formed at the tip of a stainless-steel needle and analyzed by image capture and subsequent software processing to calculate surface tension [13].

#### Rheological analysis

The rheological behavior of the ME formulations was evaluated using an MCR-302 rheometer (Anton Paar) equipped with a cone-plate geometry (50 mm diameter, 1° angle, and 96 µm gap). Samples were subjected to a shear rate ranging from 0.1 to 100 s^−1^ at both room temperature (25 °C) and skin temperature (32.5 °C) [13]. All measurements were carried out in triplicate.

#### Microemulsion stability tests

The physicochemical stability of the optimized microemulsion and its gelled form was monitored over 60 days at room temperature (25 ± 2 °C). Stability parameters included visual appearance (e.g. signs of haze, creaming, or phase separation), hydrodynamic diameter distribution, and pH. The pH was measured using a pre-calibrated PG 2000 pH meter (Gehaka, São Paulo, Brazil). Due to its high viscosity, MEgel samples were diluted with purified water (1:40 w/w) prior to analysis. All tests were conducted in triplicate.

### In vitro cytocompatibility

The cytocompatibility the ME was evaluated using the MTT [3(4,5-dimethylthiazol-2-yl)-2,5-diphenyltetrazolium bromide] assay. Assays were performed on murine macrophage cells (RAW 264.7 TIB-71^™^) and human umbilical vein endothelial cells (HUVEC, C12203).

RAW cells were cultured in Dulbecco’s modified eagle medium (DMEM, Sigma-Aldrich, D0822) supplemented with 10% (v/v) fetal bovine serum. HUVEC cells were maintained in endothelial cell growth medium (Sigma-Aldrich, C22110). Cells were seeded in 96-well plates at densities of 3 × 10^4^ (RAW) and 1.9 × 10^4^ for (HUVEC) cells/well and incubated for 24 h at 37 °C in a humidified atmosphere with 5% (v/v) CO_2_ before treatment.

Cells were then treated with varying concentrations of ME (0.1 to 10 mg/mL) for 24 or 48 h (*n* = 3 plates, each with 3 wells per concentration). Compatibility was assessed by mitochondrial metabolic activity, based on the conversion of MTT salt into insoluble formazan crystals. After incubation, formazan crystals were solubilized in DMSO, and absorbance was measured at 570 nm with a reference at 630 nm using a microplate reader (EL 800, Bio-TEK Instrument, Inc^®^, France). Cell viability was calculated normalizing untreated controls as 100%, according to the equation:


[5] $$\text{Mitochondrial activity}\left(\%\right)=\frac{{\text{OD}}_{570-630}\text{sample}}{{\text{OD}}_{570-630}\text{control}}\times 100.$$

### Statistical analysis

Statistical analyses, excluding those related to DoE, were performed using the Prism^®^ 10 software (GraphPad, California, USA). Two-way ANOVA followed by Tukey’s pos hoc test was used to assess statistical significance. Differences were considered significant when *p* < 0.05.

## Supporting Information

File 1 Additional figures and tables.

## Data Availability

Additional research data generated and analyzed during this study is not shared.
