# Supplementary material for: Quality by design optimization of microemulsions for topical delivery of Passiflora setacea seed oil
Source: Beilstein J Nanotechnol. 2025 Nov 20;16:2116–31. doi: 10.3762/bjnano.16.146 (PMC12642947; doi:10.3762/bjnano.16.146)
Supplement: File 1 — Additional figures and tables. [file Beilstein_J_Nanotechnol-16-2116-s001.pdf]

BEILSTEIN JOURNAL OF NANOTECHNOLOGY

Supporting Information

for

Quality by design optimization of microemulsions for topical delivery of *Passiflora setacea* seed oil

Daniel T. Pereira, Douglas Dourado, Danielle T. Freire, Dayanne L. Porto, Cícero F. S. Aragão, Myla L. de Souza, Guilherme R. S. de Araujo, Ana Maria Costa, Wógenes N. Oliveira, Anne Sapin-Minet, Éverton N. Alencar and Eryvaldo Sócrates T. Egito

Beilstein J. Nanotechnol. **2025,** *16,* 2116–2131. doi:10.3762/bjnano.16.146

Additional figures and tables

License and Terms: This is a supporting information file under the terms of the Creative Commons Attribution License (https://creativecommons.org/licenses/by/4.0). Please note that the reuse, redistribution and reproduction in particular requires that the author(s) and source are credited and that individual graphics may be subject to special legal provisions.
The license is subject to the *Beilstein Journal of Nanotechnology* terms and conditions: (https://www.beilstein-journals.org/bjnano/terms)

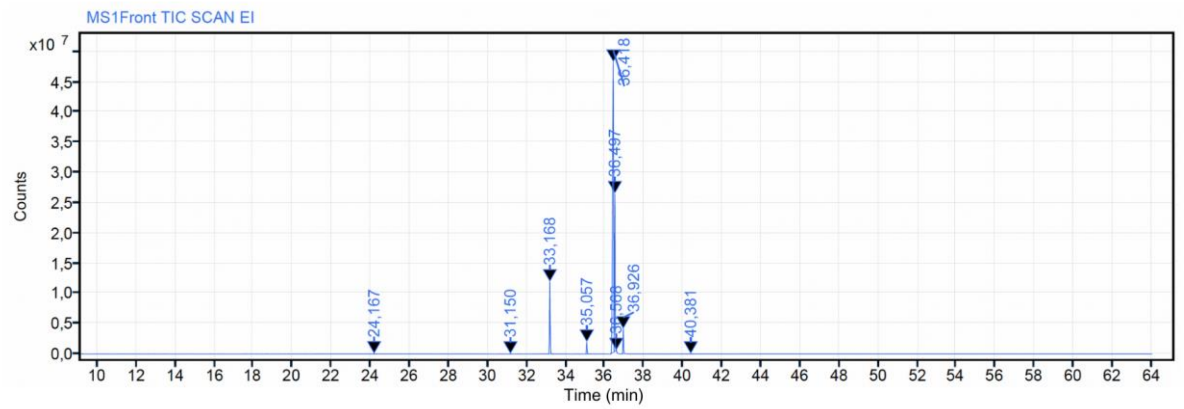

Figure S1: Gas chromatography coupled with mass spectroscopy (GC–MS) chromatogram of the constituents of transesterified *P. setacea* oil. Arrows indicate the retention time of each compound identified in Table 1.

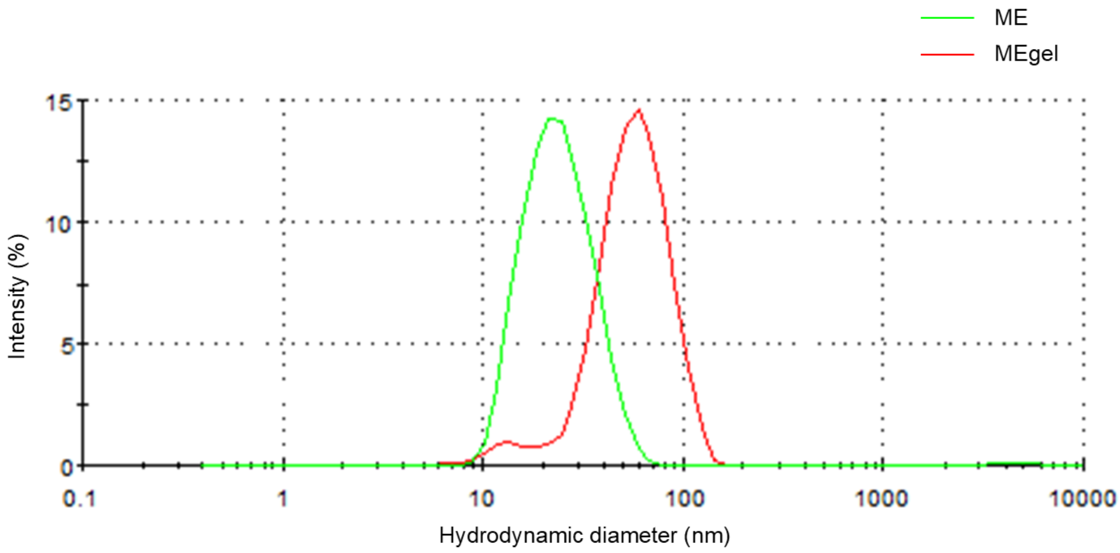

Figure S2: Hydrodynamic diameter distribution by intensity of the microemulsion (ME, green line) and its gelled form (MEgel, red line) 24 h after production.

Table S1: Risk estimation matrix by three-level interdependence scale (1: low, 3: medium, 9: high) between QTPP and CQAs.

	Droplet size	Polydispersity index (PDI)	pH	Viscosity	Visual clarity	Surface tension	Stability
**Dosage form**	9	9	1	9	1	9	1
**Route of administration**	1	1	9	9	1	1	1
**Delivery type**	3	3	1	9	1	1	1
**Appearance**	9	9	1	1	9	1	1
**Active pharmaceutical ingredient (API)**	3	3	1	1	1	1	1
**Stability**	9	9	3	1	3	1	9
**Safety**	1	1	3	1	1	1	3
**Efficacy**	9	9	1	1	1	1	1

Table S2: Risk estimation matrix by three-level interdependence scale (1: low, 3: medium, 9: high) between CQAs and CMA/CPP.

	OPS concentration	Smix ratio	Smix concentration	Vitamin E concentration	NaCMC concentration	Preservative concentration
**Droplet size**	9	9	9	3	1	1
**Polydispersity index**	9	9	9	1	1	1
**pH**	1	1	1	1	1	1
**Viscosity**	1	1	1	1	9	1
**Visual clarity**	3	3	9	1	9	1
**Surface tension**	3	9	9	1	1	1
**Stability**	9	9	9	9	3	9

Table S3: Risk estimation matrix by three-level interdependence scale (1: low, 3: medium, 9: high) between CQAs and CMA/CPP.

	Stirring speed	Stirring time	Temperature during mixing	Water addition rate	Homogenization sequence	Cooling rate after formation
**Droplet size**	9	9	9	9	3	1
**Polydispersity index**	9	3	9	9	3	1
**pH**	1	1	1	1	1	1
**Viscosity**	1	1	1	1	1	1
**Visual clarity**	3	3	3	9	3	1
**Surface tension**	1	1	1	1	1	1
**Stability**	1	1	1	9	3	1

Table S4: 2^3 Full factorial design (FFD) and their responses.

Execution order	Factor A (%)	Factor B (mass ratio)	Factor C (%)	Hydrodynamic diameter(nm)	Polydispersity index
1	15	7:3	20	35.2	0.23
2	5	9:1	10	24.5	0.29
3	5	9:1	20	14.9	0.12
4	10	8:2	15	25.6	0.29
5	10	8:2	15	26.0	0.24
6	5	7:3	20	14.1	0.21
7	15	9:1	20	37.6	0.23
8	15	7:3	10	91.4	0.20
9	5	7:3	10	23.2	0.23
10	15	9:1	10	152.0	0.24
11	10	8:2	15	25.7	0.26

Factor A: Oil concentration; Factor B: Ratio of PEG 30 castor oil and Span® 80; Factor C: Concentration of the surfactant mixture.

Table S5: ANOVA for the hydrodynamic diameter (nm) of the ME FFD.

Factor	Sum of squares	df	Medium square	F-value	p-value	Comments
Model	15952.46	6	2658.74	19.13	0.0172	Significant
A-Oil concentration	7170.63	1	7170.63	51.59	0.0056	Significant
B-Surfactants ratio	530.89	1	530.89	3.82	0.1457	Not significant
C-S_{mix} concentration	4475.05	1	4475.05	32.20	0.0108	Significant
AB	464.36	1	464.36	3.34	0.1650	Not significant
BC	2881.54	1	2881.54	20.73	0.0199	Significant
BC	429.98	1	429.98	3.09	0.1768	Not significant
Curvature	1189.19	1	1189.19	8.56	0.0612	Not significant
Residue	416.98	3	138.99			
Lack of fit	416.88	1	416.88	8888.76	0.0001	Significant
Pure error	0.0938	2	0.0469			
Total correlation	17558.63	10				

df = degrees of freedom.

Table S6: ANOVA for the ME FFD polydispersity index.

Factor	Sum of squares	df	Medium square	F-value	p-value	Comments
Model	0.0144	6	0.0024	2.70	0.2223	Not significant
A-Oil concentration	0.0002	1	0.0002	0.18	0.6983	Not significant
B-Surfactant ratio	0.0000	1	0.0000	0.02	0.8959	Not significant
C-S_{mix} concentration	0.0039	1	0.0039	4.35	0.1282	Not significant
AB	0.0008	1	0.0008	0.85	0.4234	Not significant
BC	0.0049	1	0.0049	5.51	0.1006	Not significant
BC	0.0047	1	0.0047	5.29	0.1050	Not significant
Curvature	0.0038	1	0.0038	4.24	0.1316	Not significant
Residue	0.0027	3	0.0009			
Lack of fit	0.0016	1	0.0016	2.85	0.2335	Not significant
Pure error	0.0011	2	0.0006			
Total correlation	0.0209	10				

df = degrees of freedom.

Table S7: ANOVA for the hydrodynamic diameter (nm) of the BBD of the ME.

Source	Sum of squares	df	Mean square	F-value	p-value	Comments
Model	199.52	11	18.14	401.93	< 0.0001	Significant
A-Oil conc.	49.38	1	49.38	1094.34	< 0.0001	
B-Surfac. ratio	27.48	1	27.48	608.97	< 0.0001	
C- S_{mix} conc.	24.97	1	24.97	553.32	< 0.0001	
AB	17.32	1	17.32	383.89	< 0.0001	
AC	10.91	1	10.91	241.69	< 0.0001	
BC	9.22	1	9.22	204.26	< 0.0001	
A^2	4.49	1	4.49	99.49	0.0002	
B^2	4.77	1	4.77	105.74	0.0001	
C^2	1.14	1	1.14	25.32	0.0040	
A^2C	0.3941	1	0.3941	8.73	0.0317	
AC^2	0.9575	1	0.9575	21.22	0.0058	
Residual	0.2256	5	0.0451			
Lack of fit	0.0141	1	0.0141	0.2676	0.6322	not significant
Pure error	0.2115	4	0.0529			
Total correlation	199.74	16				

df = degrees of freedom.

Table S8: ANOVA for PdI of ME BBD.

Source	Sum of Squares	df	Mean Square	F-value	p-value	Comments
Model	6.81	7	0.9732	19.41	< 0.0001	Significant
A-Oil conc.	4.16	1	4.16	83.05	< 0.0001	
B-Surfac. ratio	0.0195	1	0.0195	0.3886	0.5485	
C-S_{mix} conc.	0.5370	1	0.5370	10.71	0.0096	
AB	0.2176	1	0.2176	4.34	0.0669	
AC	0.6001	1	0.6001	11.97	0.0072	
A^2	0.9673	1	0.9673	19.29	0.0017	
B^2	0.3681	1	0.3681	7.34	0.0240	
Residual	0.4513	9	0.0501			
Lack of fit	0.2068	5	0.0414	0.6766	0.6654	Not significant
Pure Error	0.2445	4	0.0611			
Total correlation	7.26	16				

df = degrees of freedom.

Table S9: ANOVA for visual classification of ME BBD.

Source	Sum of Squares	df	Mean Square	F-value	p-value	Comments
Model	13.14	6	2.19	56.32	< 0.0001	significant
A-Oil conc.	8.00	1	8.00	205.71	< 0.0001	
B-Surfac. ratio	0.1250	1	0.1250	3.21	0.1032	
C- S_{mix} conc.	3.13	1	3.13	80.36	< 0.0001	
AB	1.0000	1	1.0000	25.71	0.0005	
BC	0.2500	1	0.2500	6.43	0.0296	
A^2	0.6405	1	0.6405	16.47	0.0023	
Residual	0.3889	10	0.0389			
Lack of Fit	0.3889	6	0.0648			
Pure Error	0.0000	4	0.0000			
Total correlation	13.53	16				

df = degrees of freedom.
